# MicroRNAs and drought responses in sugarcane

**DOI:** 10.3389/fpls.2015.00058

**Published:** 2015-02-23

**Authors:** Agustina Gentile, Lara I. Dias, Raphael S. Mattos, Thaís H. Ferreira, Marcelo Menossi

**Affiliations:** Laboratório de Genoma Funcional, Departamento de Genética, Evolução e Bioagentes, Instituto de Biologia, Universidade Estadual de CampinasCampinas, São Paulo, Brazil

**Keywords:** sugarcane, drought stress, miRNAs, transcription factors, drought tolerance, cross-species comparisons

## Abstract

There is a growing demand for renewable energy, and sugarcane is a promising bioenergy crop. In Brazil, the largest sugarcane producer in the world, sugarcane plantations are expanding into areas where severe droughts are common. Recent evidence has highlighted the role of miRNAs in regulating drought responses in several species, including sugarcane. This review summarizes the data from miRNA expression profiles observed in a wide array of experimental conditions using different sugarcane cultivars that differ in their tolerance to drought. We uncovered a complex regulation of sugarcane miRNAs in response to drought and discussed these data with the miRNA profiles observed in other plant species. The predicted miRNA targets revealed different transcription factors, proteins involved in tolerance to oxidative stress, cell modification, as well as hormone signaling. Some of these proteins might regulate sugarcane responses to drought, such as reduction of internode growth and shoot branching and increased leaf senescence. A better understanding on the regulatory network from miRNAs and their targets under drought stress has a great potential to contribute to sugarcane improvement, either as molecular markers as well as by using biotechnological approaches.

## Sugarcane: a complex grass

Sugarcane (*Saccharum* ssp.) belongs to the family *Poaceae*, subfamily *Panicoideae* and tribe *Andropogoneae*. The family contains 13 subfamilies (Sánchez-Ken et al., 2007), comprising a monophyletic clade that shows some particularities, such as the presence of a caryopsis fruit and a well-differentiated lateral embryo, a unique combination among monocotyledonous plants (GPWG, 2001). The tribe *Andropogoneae* contains species that are mostly polyploid and perennial and have a C4 photosynthetic mechanism (Clayton and Renvoize, 1982). It is one of the largest tribes of the *Poaceae* family and is widely distributed in tropical and subtropical regions of the world (Clayton and Renvoize, 1982; Sánchez-Ken and Clark, 2010).

Sugarcane, a crop of great worldwide economic importance, is responsible for approximately 75% of the global sugar production (Commodity Research Bureau, 2015), and is becoming increasingly relevant in the production of renewable energy. Sugarcane is a unique crop regarding the ability to accumulate sucrose, that can reach levels up to 50% of dry weight in its stalks (Botha and Black, 2000). Brazil stands out as the main producer of sugarcane, with a cultivated area estimated to be approximately 8.8 million hectares (as of the 2013/2014 harvest), producing more than 600 million tons, of which 46% was used for sugar production and 54% was used for ethanol production (Conab, 2013).

The cultivars that are grown worldwide are multi-species hybrids resulting from classical breeding, known as *Saccharum* spp. The species *Saccharum officinarum* was the basis for breeding programs, due to the high levels of sucrose in its stem, but this cultivar has low levels of resistance to diseases. *S. spontaneum* is another important species in the breeding program, mainly due to its characteristics of strength and resistance to pests (Miranda et al., 2008).

Sugarcane has one of the most complex genomes of all cultivated plant species, with a high chromosome number and high degree of aneuploidy (D'Hont et al., 1996). The modern hybrids have between 100 and 120 chromosomes; 70–80% of the chromosomes belong to *S. officinarum*, 10–23% of the chromosomes originated from *S. spontaneum*, and 8–13% of chromosomes are derived from recombination between the species (Piperidis et al., 2010). Despite this complexity, sugarcane has been subjected to genetic mapping using several types of molecular markers (Cordeiro et al., 2001; Ming et al., 2002a), which enabled the identification of QTLs, principally related to sugar yield, cane yield, fiber content and sucrose content (Ming et al., 2002b,c; Pastina et al., 2012). Comparative mapping between sugarcane and sorghum (*Sorghum bicolor*), another C4 grass, showed high synteny between the genomes, with 84% of the loci mapped using 242 probes presenting homology between these species, indicating their high conservation, that reaches 95.2% of sequence identity in the coding region (Ming et al., 1998; Asnaghi et al., 2000; Dillon et al., 2007; Wang et al., 2010b). Because the sugarcane genome has not been sequenced until now, sorghum has become a good reference genome for analyses involving sugarcane due to the high gene identity found between these two species (Jannoo et al., 2007; Wang et al., 2010b). Determining the genome sequence in complex organisms such as sugarcane is a complex task mainly due to the presence of a large fraction of repetitive DNA, that makes genome assembly very difficult (Setta et al., 2014). Therefore, the sequence of the sorghum genome can be used as reference, to guide the assembly of sugarcane sequences, providing a working draft of the entire sequence. Moreover, due to the high sequence identity between sugarcane and sorghum, it is possible to align to the sorghum genome partial sequences from sugarcane, allowing, for example, to identify the putative sequences of an entire mRNA or even miRNA precursors. Even though, the need of a reference genome from sugarcane is essential. This would allow, for example, to identify promoter regions, that usually are less conserved between species, and in the case of miRNAs, to clone the entire miRNA precursors. Also, as the sequencing costs decline, soon the sequencing by genotyping strategy will be feasible. This will allow the identification of single nucleotide polymorphisms (SNPs) that can be associated to several useful agronomical traits, generating new molecular markers for sugarcane breeding programs (Setta et al., 2014).

## Drought stress in sugarcane

Sugarcane development can be divided into four stages: germination, tillering, grand growth and maturity (Gascho and Shih, 1983). Each of these stages is affected in different ways by water stress. Because a reduction in growth rates is one of the conserved evolutionary responses that plants activate in response to drought, the tillering and grand growth phases are known to be critically affected by water scarcity (Ramesh, 2000; Inman-Bamber and Smith, 2005). Unfortunately, up to 80% of sugarcane yield is produced during these two phases (Singh and Rao, 1987). The sugarcane crop cycle, from cane planting and harvest of mature cane, usually takes from 12 to 18 months. A plant crop refers to the plants grown for the first time after planting. Once the above ground plant part is harvested, it will regrowth, giving rise to a new plant that will be harvest again. This cycle usually has one plant crop and 3 to 4 ratoon (regrowth) crops. Typically, farmers have about 20% of their area with “new” plants (i.e., cane that will be harvest for the first time). Therefore, unlike other crops with shorter life cycle, such as maize, soybean and wheat, sugarcane farmers do not have any flexibility to avoid dry seasons, because sugarcane will face climate up and downs that take places along the entire year.

Drought causes several effects in sugarcane. There is evidence that stomata closure, intended to reduce water loss, is triggered by a combination of the water status of adjacent cells, intensity of photon flux (Assmann and Grantz, 1990) and the water deficit sensed by roots (Smith et al., 1999). As expected, drought reduces transpiration and photosynthesis and increases leaf temperature (Rodrigues et al., 2009, 2011; Graça et al., 2010). Sugarcane cultivars differ in their responses to drought stress. Usually, the assays to infer the tolerance to drought are done using different cultivars that are ranked according to their yield under drought stress (Kumar, 2005; Silva et al., 2007; Ribeiro et al., 2013). Stalk yield and the content of soluble solids in the stalk juice usually are the key parameters used by breeders to classify the degree of tolerance to drought in sugarcane genotypes. This is because sugarcane productivity is based in these two indexes. Therefore, although most cultivars show decreased yields under drought, some are more affected than others. Interestingly, a cultivar considered as sensitive to drought, i.e., reduced yield under drought stress, may be considered a useful cultivar. For example, Ribeiro et al., found that cultivar IACSP86-2042 had a 50% reduction in stalk yield under drought stress, much higher than IACSP94-2094 (29% reduction) and SP87–365 (no reduction). However, the absolute stalk yield of these cultivars were similar under drought conditions; i.e., IACSP86-2042 had a much higher productivity under non-stressful conditions (234% higher than SP87–365 and 50% higher than IACSP94-2094).

Losses due to drought are not unusual and almost every year, some sugarcane growing regions suffer mild to severe water shortages, as has been reported in Brazil (Table 1). Therefore, drought can cause major economic losses for sugarcane growers. Interestingly, a mild drought stress can have a positive impact on sugarcane yield. It is a common practice namely in countries that use irrigation, to apply a period of drying off (water withheld) at the end of the season. The drying off period has several benefits: save water and therefore costs associated with irrigation, reduces soil compaction during harvest and may even increase sucrose content (Robertson and Donaldson, 1998; Singels et al., 2000; Inman-Bamber, 2014). The increase in sucrose content may be due to the fact that growth is more affected than photosynthesis and therefore, assimilated CO_2_ can be diverted from leaf and culm growth to sucrose accumulation in the culm. Therefore, the regulation of sugarcane responses to drought certainly will have differences with those observed from other crops and model plants.

**Table 1:** **Estimated losses in sugarcane fields due to drought stress**.

**Year**	**Region**	**Losses**	**Rain (mm H_**2**_0)/ percentage of the expected rain**
2008	São Paulo State	6.3% (Castro, 2008)	419.5/49%
2010	Zona da Mata (Pernambuco State)	40% (Cavalcanti, 2010)	300/50%
2012	Alagoas State	20% (Agẽncia Globo, 2012; Sindaçucar, 2012)	774/48.6%
2012	Pernambuco State	35% (Associação dos fornecedores de cana de pernambuco, 2012; Camarotto, 2012)	629.4/50.7%
2013	Paraiba State	30% (G1 Agency, 2012; Silva, 2013)	n.a./up to 58.7%
2013	Zona da Mata (Pernambuco State)	25% (Brasilagro, 2013)	821/48.7%
2014	Ribeirão Preto (São Paulo State)	15% (Palhares, 2014)	480/51.6%

## MicroRNAs

In addition to having conserved functions that extend beyond development, microRNAs (miRNAs) play crucial roles in the regulation of plant responses to several stimuli (Bartel, 2004), acting like a buffer for plant molecular dynamics. miRNAs are a class of small, non-coding RNAs of approximately 21 nucleotides in length that are endogenous to both plants and animals (Bartel, 2009; Carthew and Sontheimer, 2009) and function to regulate gene expression by sequence-specific interaction with target mRNAs (Bartel, 2004; Chapman and Carrington, 2007). Conserved miRNAs mainly regulate transcription factors involved in basic functions, such as cell division, hormonal control or meristem development (Garcia, 2008). miRNAs arose from genome duplications and rearrangements and, for this reason, frequently have many copies (Voinnet, 2009). However, some recent miRNAs are thought to be represented by single-copy genes that are not conserved in phylogenies (Zhang et al., 2008b).

In plants, most MIR genes possess their own transcriptional unit and are transcribed by RNA polymerase II (Pol II) into a primary miRNA (pri-miRNA) (Lee et al., 2004). The pri-miRNA forms an imperfect foldback structure, ranging from hundreds to thousands of bases (Zhang et al., 2009). These structures are stabilized by the addition of a 5′ 7-methylguanosine cap and a 3′ polyadenylated tail (Jones-Rhoades and Bartel, 2004; Xie et al., 2005; Zhang, 2005).

In nuclear processing centers called D-bodies (or SmD3/SmD3-bodies), the pri-mRNA is processed into a stem-loop precursor (pre-miRNA) and generates a double-stranded RNA duplex via a Dicer-like protein (DCL), a nuclear RNase III-like enzyme, and two RNA-binding proteins named HYPONASTIC LEAVES1 (HYL1) and the C_2_H_2_-zinc finger protein SERRATE (SE) (Kurihara et al., 2006; Lobbes et al., 2006; Fang and Spector, 2007).

The pre-miRNAs range from 60 to >400 nt in size (Xuan et al., 2011), and the double-strand RNA duplex, also called miRNA/miRNA*, ranges from 19 to 24 nt long (Reinhart et al., 2002; Bartel, 2004). The precise release of miRNA duplexes from the pre-miRNAs is both structure- and sequence-dependent (reviewed by Naqvi et al., 2012; Rogers and Chen, 2013), while the size is dependent on the action of the DCL family member (Margis et al., 2006); DCL1 produces small RNAs of 18–21 nt, while those of DCL2, DCL3, and DCL4 are 22 nt, 24 nt, and 21 nt, respectively (Voinnet, 2009). In plants, most miRNAs are processed by DCL1 (Reinhart et al., 2002) and are predominately 21 nt long (Chen et al., 2010).

The short double stranded RNAs (dsRNAs) that result from DCL processing have 2-nt 3′ overhangs and are methylated at their 3′ ends by the methyltransferase HEN-1 (Yu et al., 2005; Fang and Spector, 2007). This step protects dsRNAs from uridylation and subsequent degradation (Li et al., 2005).

The exact form in which miRNA/miRNA^*^ duplexes are transported across the nuclear membrane is unclear. In the cytoplasm, one of the strands, called the mature miRNA, is incorporated into an Argonaute protein (AGO) to form the RNA-induced silencing complex (RISC), and the miRNA* strand is usually degraded (Reinhart et al., 2002; Bartel, 2004; Voinnet, 2009).

Similar to the DCL family, the Argonaute family also has several members, and AGO1 is generally associated with miRNA biogenesis (Vaucheret, 2008). The process of choosing the miRNA strand that incorporates the complex is dependent on the thermodynamic stability of the 5′ portion of the duplex; the strand with the lower stability is incorporated by AGO1 (Eamens et al., 2009). The incorporated mature miRNA guides the RISC to scan the cytoplasm to find a specific target mRNA by base pairing, leading to mRNA cleavage or translational repression (Bartel, 2004). Therefore, in most cases, miRNAs will reduce the expression of their target mRNAs.

## miRNAs modulated by drought in sugarcane

There are several miRNAs that have been identified in a wide array of species, but only a few studies have been performed to identify the mature miRNA sequences and analyze their expression in response to drought stress in sugarcane (Ferreira et al., 2012; Thiebaut et al., 2012; Gentile et al., 2013). Sugarcane is a complex polyploid and until now its genome sequence has not been obtained. Therefore, unlike model species with sequenced genomes such as *Arabidopsis thaliana*, miRNA characterization studies are much more complicated. For example, miRNA precursors are highly unstable, making their detection in the sugarcane EST collection (Vettore et al., 2003) very difficult. A sequenced genome would facilitate the discovery of these precursors. This is particularly relevant for the discovery of novel miRNA, since the finding of a precursor is a pre-requisite to consider a new sequence as a miRNA. Similarly, the discovery of miRNA targets is greatly facilitated when the complete genome is available. Therefore, the use of the genome of sorghum, a closely related species as mentioned above, is a key strategy to overcome this limitation.

Even though, in one study (Thiebaut et al., 2012), eight sugarcane cultivars were classified into two groups based on their tolerance to drought. Plants were grown in a greenhouse for three months and then submitted to drought stress by withholding irrigation for 24 h. Although the number of detected miRNAs was higher in the more tolerant cultivars, no miRNA was found to be induced by drought under these conditions (Thiebaut et al., 2012).

In the other two works, two sugarcane cultivars that differ in their tolerance to drought stress, RB867515 (higher tolerance, HT) and RB855536 (lower tolerance, LT), were either grown in a greenhouse for three months and then kept without water for 2 or 4 days (Ferreira et al., 2012) or field-grown for 7 months under irrigation or without irrigation (rainfed) (Gentile et al., 2013). Thirteen families of mature miRNAs were found in the two sugarcane cultivars studied (Table 2).

**Table 2:**
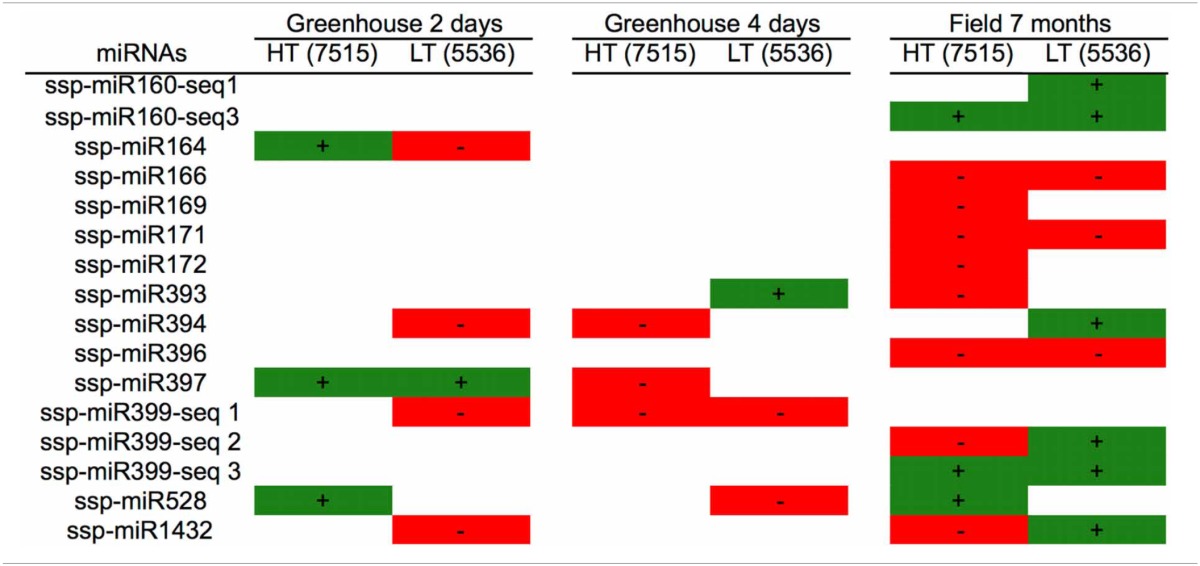
**miRNAs identified in two sugarcane cultivars differing in their tolerance to drought stress**.

RB867515 (higher tolerance, HT) and RB855536 (lower tolerance, LT), were either grown in a greenhouse for 3 months and then without water for 2 or 4 days or field-grown for 7 months under irrigation or without irrigation (rainfed). The different miRNAs were marked as induced by drought (a green box with a + sign) or repressed by drought (a red box with a − sign).

Different expression profiles of the miRNAs were observed, depending on the cultivar, the growth conditions and the type and duration of stress. Some miRNAs were found only in plants that grew in the greenhouse (ssp-miR164, ssp-miR397 and spp-miR399-seq1), while others were found only in field-grown plants (ssp-miR160-seq1, ssp-miR160-seq3, ssp-miR166, ssp-miR169, ssp-miR171 and ssp-miR172). This most likely reflects differences in the growth conditions, with field grown-plants giving a better picture of the real growth conditions that plants face in nature.

However, some miRNAs had opposing expression profiles depending on the cultivar, such as ssp-miR164, ssp-miR399-seq2 and ssp-miR1432 (up-regulated by drought in one cultivar and down-regulated in the other). In the experiment conducted in the field, a higher number of miRNAs were modulated by drought, and many of them showed a repressed profile (ssp-miR166, ssp-miR169, ssp-miR171 and ssp-miR172, among others), while others changed from down-regulated to up-regulated (spp-miR399-seq2 and ssp-miR1432) depending on the cultivar.

The timing of stress also affected the miRNA expression profile. For example, comparing greenhouse-grown plants that were stressed for 2 or 4 days, ssp-miR397 showed an altered expression profile, changing from induced to repressed, while ssp-miR399-seq1 remained invariantly down-regulated. The other miRNAs found in the greenhouse-grown plants had variable expression profiles, without a specific pattern. After 7 months, five miRNAs from the rainfed field-grown plants presented the same profile in both cultivars; two were induced (ssp-miR160-seq3 and ssp-miR399-seq3) and three were repressed (ssp-miR166, ssp-miR171 and ssp-miR396). Only two miRNAs (ssp-miR399-seq2 and ssp-miR1432) had opposite profiles among the different cultivars.

**Figure 1:**
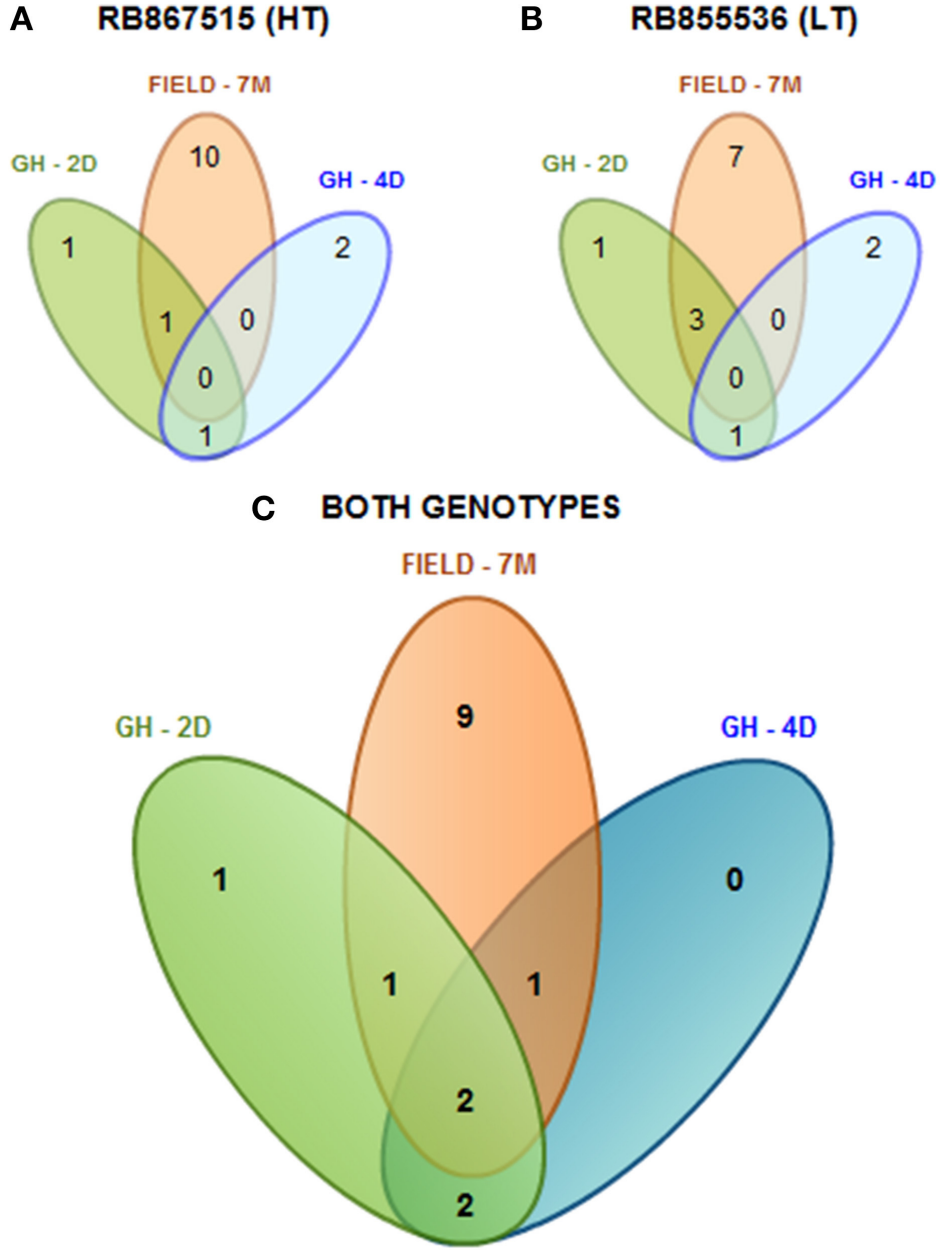
**Diagram of all the differentially expressed mature miRNAs found in sugarcane**. miRNAs found among the different stress times (2 days, 4 days, and 7 months) under greenhouse (GH) and field-grown (FIELD) conditions in the more tolerant cultivar (RB867515, HT) **(A)**, in the less tolerant cultivar (RB855536, LT) **(B)** or in both genotypes together **(C)**.

As a summary, by evaluating two sugarcane cultivars that differ in their level of drought tolerance according to their performance under field conditions (Gentile et al., 2013), a total of 16 mature miRNAs were found (Figure 1). Among the cultivars, we found that 15 mature miRNAs were differentially expressed and identified in the cultivar with higher tolerance to drought (HT, Figure 1A), while 14 were found in the cultivar with lower tolerance (LT, Figure 1B). We found that only two miRNAs (spp-miR394 and ssp-miR528) were shared among the different stress durations (2 days, 4 days, and 7 months) and two growing conditions (greenhouse and field) (Figure 1C).

It is remarkable that the expression patterns of the majority of the miRNAs did not display clear correlations with the differences in drought tolerance observed in the two sugarcane cultivars. Only ssp-528 presented a consistent induction in the RB867515 cultivar, which has high tolerance to drought (Table 2). The miRNA expression profiles were influenced by the genetic background from the distinct sugarcane cultivars, and this was more evident under greenhouse conditions. These data suggest that miRNAs do not fully explain the different levels of drought tolerance observed in the sugarcane cultivars.

Several studies in other plants species also have identified miRNAs that are modulated by drought (Table 3). Until now, the majority of the miRNAs associated with this stress were induced by drought. Rice (*Oryza sativa*) is the plant with the largest number of identified miRNAs that are modulated by drought (35 miRNAs, Tables 3, 4). Barley (*Hordeum vulgare)* was the plant with the lowest number of identified miRNAs related to drought (4 miRNAs, Table 3). Bean (*Phaseolus vulgaris)* was the only plant species that had only induced miRNAs (6 miRNAs).

**Table 3:**
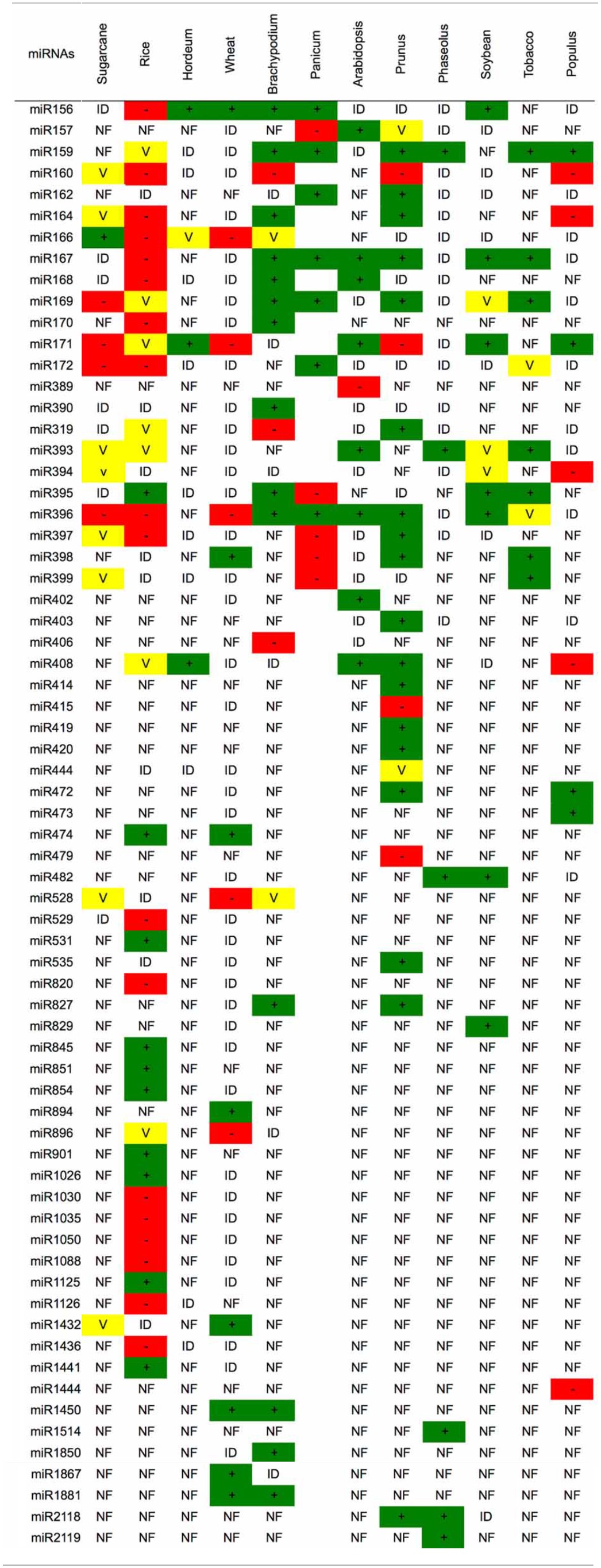
**miRNAs identified in several plant species under drought stress**.

In the table, the different miRNAs were marked as induced by drought (a green box with a +), repressed by drought (a red box with a −), variably induced (a yellow box with a V), simply identified but not differentially expressed (ID) or NF (not found). No information is available for the miRNAs boxed with no mark in Panicum. The variable induction (V) is explained further the bottom of the table, depending on the cultivars, treatments, tissue or miRNAs species analyzed. References - Sugarcane: Thiebaut et al. (2012), Ferreira et al. (2012), Gentile et al. (2013); Rice: Zhao et al. (2007), Sunkar et al. (2008), Shen et al. (2010), Zhou et al. (2010), Shaik and Ramakrishna (2012), Xia et al. (2012), Mutum et al. (2013); Hordeum: Kantar et al. (2010); Wheat: Kantar et al. (2011); Brachypodium: Budak and Akpinar (2011); Bertolini et al. (2013); Panicum: Sun et al. (2012); Arabidopsis: Sunkar and Zhu (2004), Liu et al. (2008), Chen et al. (2012), Li et al. (2012); Prunus: Eldem et al. (2012); Phaseolus: Arenas-Huertero et al. (2009); Soybean: Li et al. (2011b), Ni et al. (2012); Ni et al. (2013); Tobacco: Frazier et al. (2011); Populus: Li et al. (2011a), Ren et al. (2012), Shuai et al. (2013).

**Table 4:**
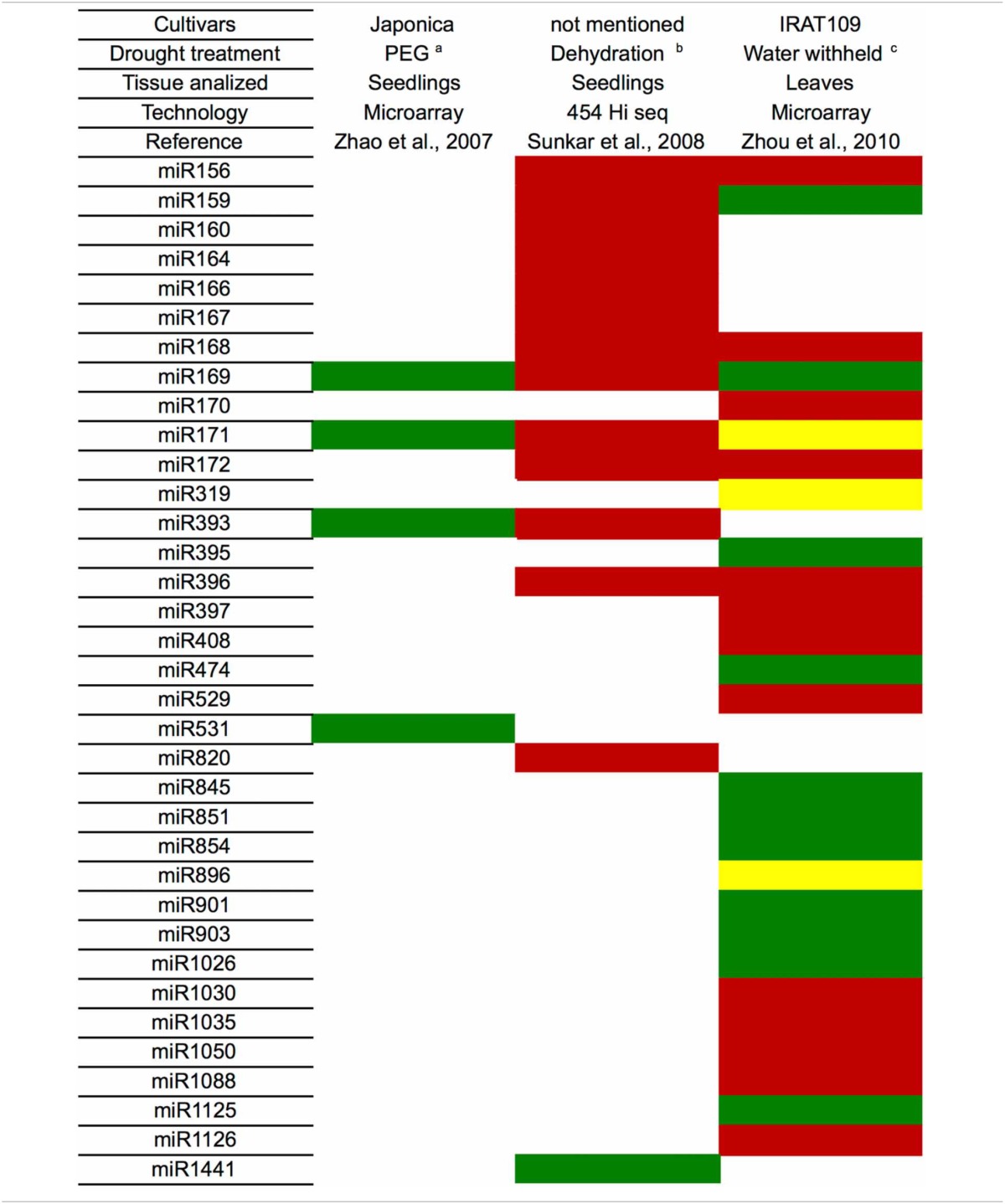
**miRNAs identified under drought stress in *Oryza sativa***.

In the table, the different miRNAs were marked as induced by drought (a green box), repressed by drought (a red box), variably induced (a yellow box). ^a^15% PEG: 0, 0.5, 2, 6, 24, and 48 h; ^b^Dehydrated for 12 h; ^c^In pots: at tillering stage, water was withheld. Collected samples at 8, 10, 12, and 14 DAW (days after water withheld). At flowering stage, collected at 5 and 6 DAW.

However, similar to the results in sugarcane, the expression pattern for a given miRNA was variable depending on the genetic background (species), type of drought treatment (PEG, dehydration, mannitol), tissue (leaves, seedlings, spikelets, roots), cultivar and growth condition (greenhouse, field, hydroponic). The miRNA that was differentially expressed in the most species was miR396 (9 species), followed by miR171, which is present in 8 plant species (Table 3). However, no miRNA was always induced or repressed in all the plant species analyzed. Moreover, no miRNA was differentially expressed in all plant species. Interestingly, sugarcane had the most variable miRNA expression profile, most likely reflecting the different cultivars, treatments and tissues that have been analyzed.

## Sugarcane miRNA targets

Plant miRNAs directly affect their target genes by nearly perfect base pairing complementarity, leading to cleavage or translation repression of these genes. Plant microRNAs can be classified into several different families, and the members of each family have very similar mature sequences. It has been reported that conserved miRNAs from the same family may have the same target genes in different species, as shown in the two model plants *Arabidopsis thaliana* and *O. sativa* (Cuperus et al., 2011). Combining data sets from high-throughput sequencing studies, Cuperus et al. (2011) identified eight miRNA families with a common ancestry in all embryophytes and a range of other families that share similar members between eudicots. The high similarity between the miRNA species in different plants allowed the development of tools for the prediction and validation of the target genes that are regulated by these miRNAs.

Several freely available tools are dedicated to the prediction of miRNA targets in plants and have been widely used (Rhoades et al., 2002; Jones-Rhoades and Bartel, 2004; Fahlgren and Carrington, 2010). Ready-to-use online tools include RNAHybrid (Krüger and Rehmsmeier, 2006), UEA sRNA-tools (Moxon et al., 2008), Target-Align (Xie and Zhang, 2010), psRNAtarget (Dai and Zhao, 2011) and PMTED (Sun et al., 2013). All of these use algorithms that rely mostly on sequence complementarity analysis, implementing filtering criteria that aim to simulate the target recognition process that occurs within the RISC complex (Bartel, 2009). The first algorithms that was developed for target identification in plants required only that the miRNA:mRNA alignment did not exceed four mismatches, regardless of the position or nature of those mismatches. Recently, more sophisticated approaches have been successfully increasing target prediction results in several plant species, including sugarcane (Zanca et al., 2010). They were developed using a scoring system that differentiates gaps, simple mismatches, G:U pairing and consider the position at which these features occur in the alignment (Zhang et al., 2006). However, each of these tools uses different implementations of these criteria, which may be a source of divergent results.

The proliferation of target prediction tools over the last 10 years makes the choice and evaluation of their results a challenge. A recent study, by Srivastava et al. (2014), showed how 11 plant miRNA target prediction tools compare to each other. Most of the tested tools use modifications of the Smith-Waterman alignment algorithm (which is very precise, with low computational cost for short sequences). The best ranked tools make their predictions by coupling alignment results with programs that access secondary structure of RNA molecules and parameters based on the most recent findings on miRNA:Target recognition. The fact that all of the plant-specific tools have been developed and trained using Arabidopsis miRNAs explains the high overlap between their analysis, and also the lower success rate in predicting miRNA targets in non-model organism. Non plant-specific algorithms present large number of predictions with very low precision.

The results described by Srisvastava et al. also show that, although still highly skewed toward Arabidopsis, a number of tools perform well when predicting targets from other plant species, given that the user sets optimized parameters for the analysis. The most reliable and rapidly obtained results were observed with Targetfinder (Fahlgren et al., 2007), psRNAtarget (Dai and Zhao, 2011) and TapirHybrid (Bonnet et al., 2010).

It is noteworthy that algorithms that use prediction criteria beyond the concept of high sequence complementarity are amongst the best performers. But, as highlighted by the authors, the occurrence of false negatives suggests there are important target recognition details still to be uncovered. Therefore, when working with species other than Arabidopsis, users are advised to avoid default settings of those tools. The recommended approach is to use an experimentally validated dataset as control, from the same species or the closest relative, to adjust the parameters of the algorithm.

The degradation rate of a particular miRNA seems to be highly dependent on its target abundance and complementarity. After the cleavage of a target, the miRISC (miRNA and RISC complex) must survive; if this complex is not maintained, the released miRNA could form another miRISC, and another round of targeting would occur (Meng et al., 2011). Because the induction or repression of a particular miRNA may depend on stresses and cell type, it is expected that additional non-conserved miRNAs will be discovered as experiments with a wide array of conditions are performed.

Few studies have presented data on the expression of target genes in sugarcane (Zanca et al., 2010; Ferreira et al., 2012; Thiebaut et al., 2012; Carnavale-Bottino et al., 2013; Gentile et al., 2013; Ortiz-Morea et al., 2013), and only a fraction of these are related to drought stress (Ferreira et al., 2012; Thiebaut et al., 2012; Gentile et al., 2013). In fact, in most cases, researchers rely on RT-qPCR expression pattern analysis of possible targets that were previously predicted by *in silico* tools. Basically, these tools analyze the complementarity between a miRNA and a transcript and calculate the unpaired energy (UPE) that would be necessary to open the secondary structure around the small RNA target site on the mRNA (Zanca et al., 2010; Dai and Zhao, 2011). A recent approach for target validation is 5′ RLM-RACE (Llave et al., 2011), which provides the amplification of the 3′ cleavage product from the miRNA:target interaction events. However, the validation of the cleavage of miRNA targets under drought stress by this approach in sugarcane has not yet been reported. It is worth noting that miRNAs may interfere with gene expression by causing mRNA cleavage or by blocking mRNA translation.

In spite of the wide array of bioinformatics tools that can be used as first approach to identify putative targets, few reports in humans, animals and plants have used experimental tools to further prove the true targets of a particular miRNA. It is beyond the scope of this review to discuss the experimental strategies that can be used as well as their limitations, since this has been addressed by several reviews on this subject (Thomson et al., 2011; Ding et al., 2012; Moqadam et al., 2013). In general, a first approach is to check if the miRNA::target pair has opposed expression patterns, i.e., when the miRNA is up-regulated, the target is down regulated and vice-versa. In another approach, a construct containing the miRNA binding site in the coding region of the luciferase gene or in the 3′UTR is co-expressed with another construct overexpressing the miRNA. Decreased levels of luciferase can be observed in those cases where a true miRNA::target interaction takes place, as observed by Liu et al. (2014). The cleavage site in the mRNA can also provide an experimental evidence of miRNA action, by using RNA ligase mediated- 5′ rapid identification of cDNA ends (5′ RLM-RACE), as firstly observed for miR171 and a member of the Scarecrow-like (SCL) transcripton factor (Llave et al., 2002).

However, these and several other strategies aiming a single miRNA::target pair are work intensive. They are not suitable to address the challenge of evaluating the high number of miRNA::targets interactions predicted in miRNA expression profiling methods using DNA chips or RNAseq. A straightforward method to address this challenge is the analysis of the degradome. This strategy allows the sequencing of the entire set of cleavage products derived from all miRNAs in a sample, allowing the mapping of the exact cleavage site (Addo-Quaye et al., 2008; German et al., 2008). Clearly, with the decreasing costs of DNA sequencing, the research on sugarcane miRNAs will soon benefit from these high throughput technologies.

## miRNAs and sugarcane responses to drought

The miRNA expression patterns and the predicted targets described in the previous works with sugarcane under greenhouse and field conditions (Ferreira et al., 2012; Gentile et al., 2013) provide a working model of the defense strategies that might be regulated by miRNAs in sugarcane exposed to drought.

Plants grown under field conditions had increased levels of ssp-miR166 when stressed by drought. This miRNA targets transcription factors from the homeobox-leucine zipper. The overexpression of a transcription factor from this family caused reduced internodes in the model plant *Arabidopsis thaliana*. Interestingly, reduced stalk length is one of the most remarkable phenotype in sugarcane plants exposed to drought (Inman-Bamber and Smith, 2005; Silva et al., 2008, and references therein). Therefore, reduced ssp-miR166 would increase the levels of the transcription factor that is involved in shortening the internodes.

ssp-miR171, was also repressed by drought under field conditions, targets a sugarcane gene encoding a protein with high identity to members of the scarecrow-like transcription factor (SCL—GRAS domain protein) family. The Arabidopsis homologs of this protein induce shoot branching and are targets of miR171, and overexpression of miR171 caused reduced shoot branching in transgenic plants (Wang et al., 2010a). Shoot branching is reduced under drought stress in sugarcane, compromising plant survival and reducing crop productivity (Inman-Bamber and Smith, 2005; Silva et al., 2008; Kapur et al., 2011). Decreased levels of ssp-miR171 and conversely increased levels of the SCL transcription factor, could be a sugarcane response to counteract the deleterious effects of drought on tillering.

ssp-miR160 was up-regulated in response to drought in field-grown plants. This miRNA targets a sugarcane gene that has high identity to the VNI2 protein from Arabidopsis. This protein repress the activity of a transcription factor, VASCULAR-RELATED NAC-DOMAIN7 (VND7), that is a master inducer of xylem formation (Yamaguchi et al., 2010). Although, to our knowledge, there are no works showing xylem differentiation in response to drought in sugarcane, this response has been observed in poplar trees (Arend and Fromm, 2007). Therefore, ssp-miR160 induction could lead to decreased levels of VIN2, releasing the action of VND7, that would work in xylem differentiation. This could, in turn, improve the ability of sugarcane plants to transport water.

Delaying leaf senescence is an agronomical trait that has a positive impact on plant yield under drought stress, as observed in sorghum (Borrel et al., 2000). Moreover, increased levels of isopentenyltransferase, the rate-limiting step of cytokinin biosynthesis, delay senescence in transgenic tobacco plants and increase drought tolerance (Rivero et al., 2007). Leaf senescence is observed in sugarcane plants under drought stress and is correlated with decreased crop productivity (Inman-Bamber, 2004; Lopes et al., 2011). ssp-miR399 is induced by drought in field grown sugarcane and targets a protein associated with leaf senescence in maize. Increased levels of this miRNA could be a response to keep a green leaf phenotype, allowing sugarcane plans to sustain photosynthesis for a longer period under stress.

As observed in many other species, drought induces oxidative stress in sugarcane, increasing H_2_O_2_ content and the levels of lipid peroxidation (Cia et al., 2012). A miRNA, ssp-miR169, repressed by drought in one sugarcane cultivar grown in the filed, targets a glutathione S-transferase (GST). These enzymes are involved in the detoxification of compounds generated during stress and transgenic plants overexpressing GSTs have increased tolerance to oxidative stress and water deficit (George et al., 2010; Ji et al., 2010). Reduced levels of ssp-miR169 could increase GST levels and therefore reduce the toxic effects of reactive oxygen species.

miRNA expression profiles also revealed a range of transcription factors that may be involved in plant responses and tolerance to drought stress (Table 5). We found many transcriptions factors, such as NAC domain, homeobox-leucine zipper, Nuclear Factor YA, GRAS/SCL, APETALA2 and bZIP transcription factors. All of these have been described as being related to drought stress and/or increasing tolerance to water stress when overexpressed in other plants (Dezar et al., 2005; Nelson et al., 2007; Stephenson et al., 2007; Li et al., 2008; Ma et al., 2010; Ditt et al., 2011; Golldack et al., 2011; Krishnaswamy et al., 2011). Other targets encode a wide array of proteins. A NSP-interacting kinase (NIK), which is a member of the serine/threonine kinase subfamily that is involved in plant development and responses to external stimuli. An auxin receptor that specifically binds to a repressor that is then degraded, allowing the expression of genes related to auxin, was also found (Dharmasiri et al., 2005). In addition to these targets, a GAPDH (glyceraldehyde-3-phosphate dehydrogenase), which is involved in generating more ATP, and a pyruvate dehydrogenase enzyme, involved in carbon balance during the stress (Chaves et al., 2009) were also identified. An inorganic pyrophosphatase 2-like was found among the targets and has already been reported to confer tolerance to drought stress when overexpressed in several plants (Gaxiola et al., 2001; Park et al., 2005; Zhang et al., 2011). Finally, some enzymes were identified as involved in cell modifications, such as laccases, that reduce cell elongation during drought stress (Cachorro et al., 1993). These results showed that the sugarcane miRNAs identified under drought stress could regulate different genes that function in several metabolic pathways, indicating the plasticity and the complexity of sugarcane responses to this stress.

**Table 5:** **Target prediction for the miRNAs that were differentially expressed in drought-stressed sugarcane plants**.

**MicroRNA**	**Experiment**	**Target Acc**	**GenBank Acc**	**Target Description (PS RNATarget)**
sspmiR160-seq 1	Field	SCCCLR1C04H01.g	____	NAC domaincontaining protein 68-like (*Brachypodium distachyon*)
ssp-miR164	Greenhouse	SCEPRT2048G05.g	CA138286	NAC transcription factor *(Hordeum vulgare)*
ssp-miR164	Greenhouse	SCCCAM1001A03.g	CA070971	MDR-like ABC transporter (*Oryza sativa* - Japonica Group)
sspmiR166-seq 3	Field	SCRFLR1034E12.g	CA125267	Homeobox-leucine zipper protein HOX32 (*Oryza sativa*)
sspmiR169-seq 2	Field	SCACST3157E03.g	CA180615	Nuclear transcription factor Y subunit A-10 (*Zea mays*)
sspmiR171-seq 2	Field	SCJFAD1013C10.g	CA067246.1 CA067169.1	Scl1 protein (*Oryza Sativa –* Japonica Group)
sspmiR172	Field	SCJLRT1022F08.g	CA135950.1 CA135877.1	Floral homeotic protein APETALA 2-like (*Brachypodium distachyon*)
ssp-miR394	Greenhouse	SCQGAM2027G09.g	CA086777	Glyceraldehyde-3-Phosphate dehydrogenase (*Triticum aestivum*)
sspmiR394	Field	SCUTLR1037A06.g	CA126572	Protein N5P-interacting kinase 1-like (*Brachypodium distachyon*)
ssp-miR528	Greenhouse	SCJFRT2058D11.g	CA141137	UBX domain-containing protein (*Oryza brachyantha*)
sspmiR528	Field	SCCCCL1002D10.b	CA092987	Pyruvate dehydrogenase El alpha subunit
ssp-miR397	Greenhouse	SCQSAD1056B07.g	CA067772.1 CA067688.1	Laccase-23-like (*Brachypodium distachyon*)
ssp-miR1432	Greenhouse Field	SCSFFL4085D03.g	CA244979.1 CA244895.1	ABRE-binding factor BZ-1 bZIP transcription factor1 (*Zea mays)*
ssp-miR393	Greenhouse	TC120009	CA079863 CA080651 CA173890	Auxin-responsive factor TIR1-like protein (*Populus tomentosa)*
ssp-miR399seq1	Greenhouse	SCACHR1037A06.g	CA101430	Inorganic pyrophosphatase 2-like (*Brachypodium distachyon*)
sppmiR399-seq 3	Field	SCJFLR1017A12.g	CA122207	Senescence-associated like protein (*Zea mays*)

Target Acc: the accession number in the SUCEST or SoGI databases; GenBank Acc: the accession number in the GenBank database; Target description: a description of the target according to a BLAST search of the GenBank database, including the name of the organism producing the best hit.

## Conclusions and perspectives

In this review, we have evaluated studies describing the expression profiles of miRNAs from sugarcane under drought stress. Cultivars that differ in their level of drought tolerance were grown under different conditions and stressed in different ways. Our analysis provides insights into the complexity of the sugarcane miRNA regulatory network under drought stress. Few studies have evaluated plant responses under real field conditions, and we found that these responses differ considerably from those observed in the greenhouse. The different genetic background of the cultivars used in the sugarcane studies highlight a new layer of complexity in miRNA expression. Interestingly, this complexity observed in sugarcane was also detected in other plant species. Taken together, the data from miRNA expression under drought stress suggest that plants may adjust their microtransptome in a variety of ways to cope with different phases and intensity of drought stress and that these responses may be fine-tuned in particular genetic backgrounds.

This complexity of expression patterns urges us to move toward functional assays and the use of mutants with decreased or increased expression of selected miRNAs. These mutants could be produced, for example, by overexpressing or silencing miRNA precursors in transgenic plants, and will be extremely helpful in assessing the role of miRNAs in drought responses.

### Conflict of interest statement

The authors declare that the research was conducted in the absence of any commercial or financial relationships that could be construed as a potential conflict of interest.
